# Endocrown with Leucite-Reinforced Ceramic: Case of Restoration of Endodontically Treated Teeth

**DOI:** 10.1155/2015/750313

**Published:** 2015-10-18

**Authors:** Leonardo Fernandes da Cunha, José Mondelli, Caroline Moreira Auersvald, Carla Castiglia Gonzaga, Rafael Francisco Lia Mondelli, Gisele Maria Correr, Adilson Yoshio Furuse

**Affiliations:** ^1^Graduate Program in Dentistry, Positivo University, 5300 Rua Professor Pedro Viriato Parigot de Souza, 81280-330 Curitiba, PR, Brazil; ^2^Department of Operative Dentistry, Endodontics, and Dental Materials, University of São Paulo, Bauru, Al. Octávio Pinheiro Brisolla 9-75, Vila Universitária, 17012-901 Bauru, SP, Brazil

## Abstract

A common problem encountered by dentists is the restorative treatment of nonvital teeth. When the pulp chamber presents appropriate conditions for retention, the endocrown is indicated. This monolithic, ceramic adhesive restoration is singularly used yet warrants wider recognition and use. The endocrown allows preservation of the tooth structure and is minimally invasive. Currently, this treatment option, of a core buildup and full coverage restoration, reduces tooth structure excessively. This treatment presents not only functional limitations but also aesthetic concerns. Recently, the VITA-PM9 system, a leucite-reinforced glass ceramic, has been increasingly used in a variety of clinical situations due to its satisfactory physical-mechanical and aesthetic properties. Therefore, the present study describes a case of surgical restoration of a nonvital tooth using the endocrown technique and the VITA-PM9.

## 1. Introduction

The restoration of endodontically treated teeth has long been a challenge for dentists. These teeth often have little remaining crown, and the biomechanical principles of retention and resistance are compromised [1]. One treatment option for previously treated teeth is the placement of intracanal posts to retain and anchor the restoration material [2]. However, many clinicians are unclear on the criteria for utilizing cementation of intraradicular posts as many variables must be considered [3]. In addition, placement of the intracanal post risks root perforation and thinning of the root canal walls as a result of overpreparation [4]. These cases often require preparation of the remaining or entire crown in order to stabilize the remaining tooth structure and replace the lost material. However, a simpler, faster, and more conservative alternative is needed.

In order to devise a conservative treatment option for endodontically treated teeth, Bindl and Mörmann proposed the restoration of nonvital teeth using the pulp chamber for support of the definitive onlay restoration. This all-ceramic restoration technique, called the endocrown, was first described using the CEREC system [5]; however, other systems may be employed in this particular restoration [6].

Recently, the VITA-PM9 system (Vident, Brea, CA, USA) was released, which is a pressed ceramic that uses a microparticulate coating. The system employs thin feldspathic ceramic, which provides excellent resistance and polish. The system is commercially available in tablet form and is recommended for use in anterior crowns, veneers, inlays, onlays, and partial crowns.

The present case report describes an aesthetic and conservative posterior endocrown restoration of a nonvital tooth using VITA-PM9.

## 2. Case Presentation

**Figure 1:**
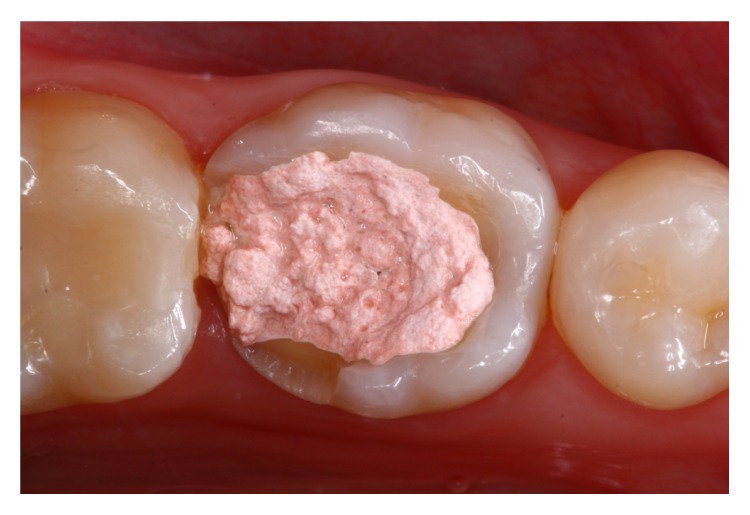
Initial examination of a 23-year-old female patient. A provisional restoration was identified within severely damaged molar (46).

**Figure 2:**
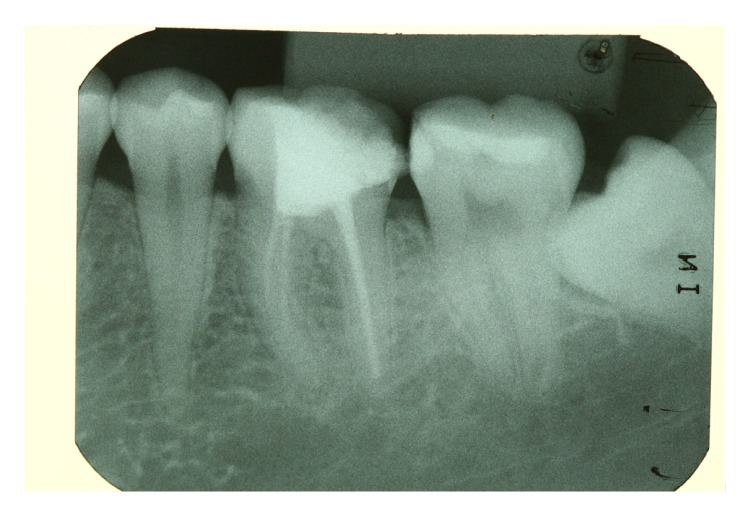
Initial radiography indicates that the molar was endodontically treated.

**Figure 3:**
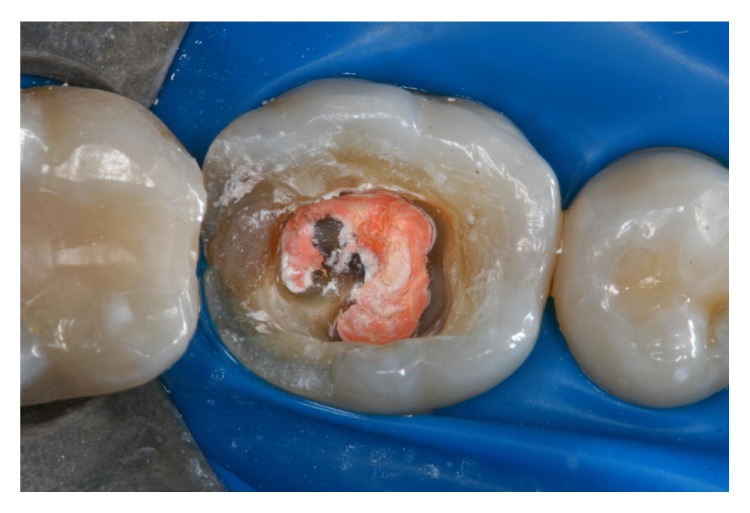
The molar after removal of the provisional restoration.

A 23-year-old female patient presented for evaluation at the Department of Restorative Dentistry, Bauru School of Dentistry, University of São Paulo, Brazil. Radiographic and clinical examinations were performed initially (Figures 1 and 2), and a provisional restoration of a nonvital molar tooth (46) was identified. After removing the provisional restoration, an endocrown restoration was recommended due to the amount of remaining tooth structure (Figure 3).

**Figure 4:**
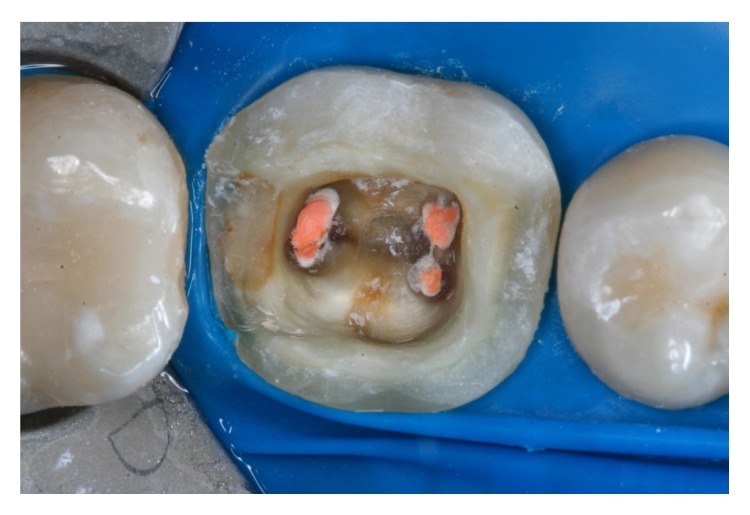
The tooth is first prepared by making the coronal pulp chamber continuous with the access cavity. The remaining tooth structure is still robust.

Shade selection was then performed. The surrounding teeth were restored with composite resin in a single section and covered with a rubber dam. The pulp chamber was exposed and the occlusal surface was prepared with a diamond bur (Mani, Japan) (Figure 4). The entire cavity and interocclusal space were evaluated. An impression was obtained using retraction cords (Pro Retract 0000, FGM; Joinville, SC, Brazil) and vinyl polysiloxane (Express XT, 3M/ESPE; Seefeld, Germany). The heavy-bodied and light-bodied impression materials were used.

A provisional restoration was constructed from a bis-acryl resin (ProTemp 4, 3M ESPE; Seefeld, Germany).

**Figure 5:**
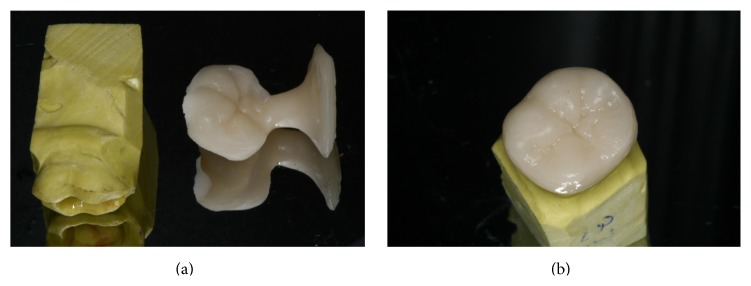
A pressed endocrown (VITA-PM9) with (a) and without (b) the attached sprue is positioned with the master mold.

**Figure 6:**
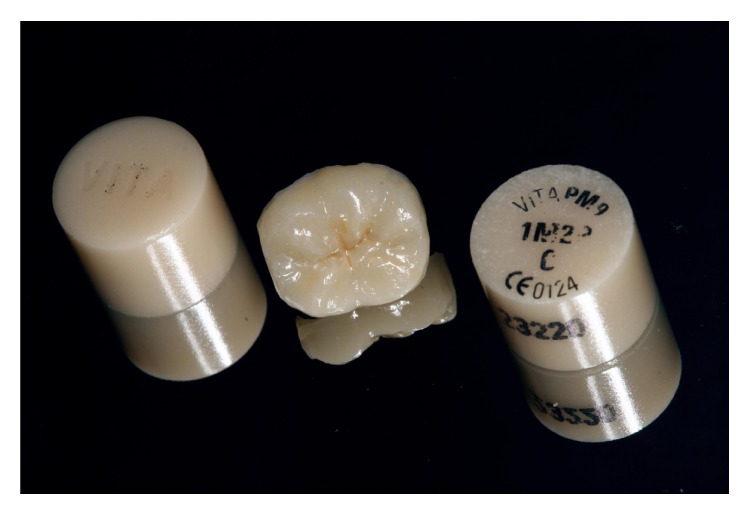
The monolithic ceramic endocrown after customization with the VITA AKZENT Plus system.

The endocrown was fabricated with an injected leucite-reinforced ceramic (VITA-PM9; Bad Säckingen, Germany) using the heat press technique (Figure 5). The VITA AKZENT Plus (Bad Säckingen, Germany) was custom-applied to enhance the shading and optical characteristics of the restoration (Figure 6).

The internal surface was etched with hydrofluoric acid (Porcelain Etchant, FGM Produtos Odontológicos; Joinville, Santa Catarina, Brazil) for 60 s, rinsed with running water, and dried with an air syringe. The restoration was treated with Prosil, which is a silane coupling agent (FGM, Joinville, Santa Catarina, Brazil) according to the manufacturer's instructions. One coat of hydrophobic resin (Adper ScotchBond Multi-Purpose, 3M/ESPE, Seefeld, Germany) was applied and light-cured for 10 s.

**Figure 7:**
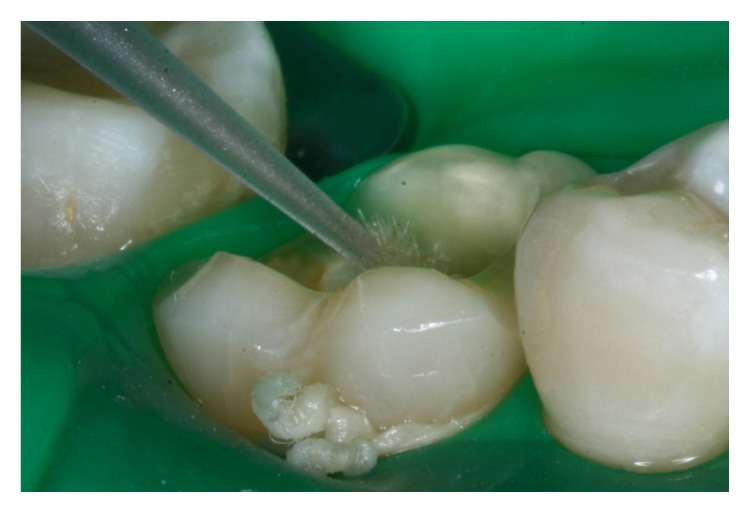
A conventional adhesive system (RelyX ARC) was used to bond the endocrown to the prepared tooth.

**Figure 8:**
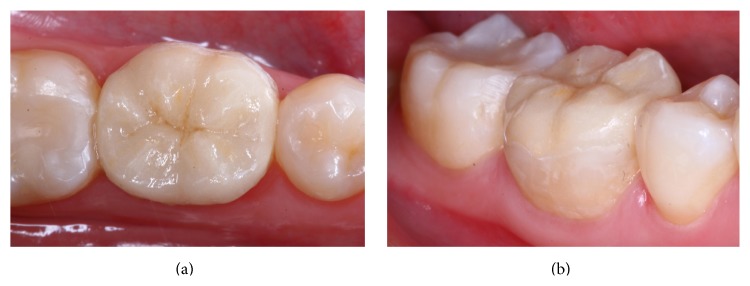
Final occlusal (a) and vestibular (b) view after bonding the endocrown using the VITA-PM9 system in a nonvital tooth.

Rubber dam was used to achieve proper isolation and then hydrophobic agent was applied onto the tooth surface (Figure 7). The endocrown restoration was bonded with a dual curable resin-based luting agent (Rely X ARC, 3M/ESPE; Seefeld, Germany). The excess cement was removed, and the restoration was light polymerized using an LED-curing unit (Radii-Cal, SDI, Victoria, Australia) for 40 seconds per margin. The restoration was examined for any occlusal interference using ceramic finishing instruments (Jota AG Rotatory Instruments, Rüthi, Switzerland). The final restoration is shown in Figures 8(a) and 8(b).

## 3. Discussion

The choice to reconstruct or restore nonvital teeth is a difficult decision for dental professionals. Aspects such as planning, selection of the restorative system, and the specific cavity preparation should be carefully considered [1].

Endodontic crowns, known as endocrowns, were initially proposed in 1999 by Bindl and Mörmann, who employed the CEREC system (Sirona) [5]. The restoration was constructed using computer-aided design/manufacturing (CAD/CAM) and bonded to the preparation with reduced macroretention. However, other systems may also be employed, and recently Lander and Dietschi used the Empress II system to restore two molars with the endocrown technique [7]. In the present report, the VITA-PM9 system was employed. This dental ceramic system, newly introduced to the market, may show long-term efficacy similar to other VITA systems [8, 9]. According to the manufacturer, the VITA-PM9 is a pressed ceramic in a fine feldspathic composition, which produces excellent aesthetic results and polish. The system is also available in a variety of colors and translucencies and can be used in a wide range of cases including inlays, veneers, anterior crowns, onlays, and partial crowns, as in the present study.

The VITA AKZENT Plus uses glazing and masking technique and glazing agents to enable shade modification by the clinician. Fluorescent masking of the stains ensures excellent shading and can be used to apply natural surface effects without requiring a ceramic layer. This monolithic ceramic restoration, according to Schultheis et al., appears to be a reliable alternative for posterior load-bearing teeth, whereas a bilayer configuration is more susceptible to low load fracture failure [10].

The quantity and quality of the coronal structure are crucial in this clinical scenario [4]. Cast metal cores or intracanal posts are frequently employed clinically. Goto et al. observed that metallic cores can generate a wedge effect on the root and require a longer laboratory phase [11]. By contrast, the adhesion of prefabricated posts has limited long-term stability [12], making this procedure effective only in selected cases. Thus, other therapeutic alternatives should be considered. When the pulp chamber retention cavity is favorable, as in the present case, endocrown restoration may be indicated to restore the biomechanics of the tooth [6].

In the present case, the loss of occlusal tooth structure was an indication for an onlay preparation, allowing thicker occlusal coverage with ceramic. Recently, a superior internal fit was observed in a study employing heat press manufacturing (VITA-PM9) compared with a CAD/CAM fabricated all-ceramic onlay [13]; both techniques provided clinically acceptable marginal and internal fit after luting. Additionally, the onlay cavity preparation was surrounded by enamel, which improved the durability of the adhesive system [14, 15]. Several factors affect the accuracy of the die, such as, impression technique, the elastic recovery of the material, and impression and die material [16]. These factors when controlled properly result in acceptable cement film thickness. Thus, no spacer was used on the die.

With the development of adhesive cementation systems, the need for macroretentive preparation for crowns has decreased [7]. These systems also balance the strength of the leucite glass ceramic crowns with the strength of a strong ceramic [17]. Hence, an endocrown composed of leucite-reinforced ceramic favors preservation of the tooth structure and is consistent with the goal of minimally invasive dentistry.

The endocrown ceramic restoration using the VITA-PM9 system is a feasible and conservative approach for mechanical and aesthetic restoration of nonvital posterior teeth.
